# Identification of Plasma Fatty Acids in Four Lipid Classes to Understand Energy Metabolism at Different Levels of Ketonemia in Dairy Cows Using Thin Layer Chromatography and Gas Chromatographic Techniques (TLC-GC)

**DOI:** 10.3390/ani10040571

**Published:** 2020-03-29

**Authors:** Enrico Fiore, Rossella Tessari, Massimo Morgante, Matteo Gianesella, Tamara Badon, Silvia Bedin, Elisa Mazzotta, Michele Berlanda

**Affiliations:** Department of Animal Medicine, Production and Health, University of Padova, Viale dell’ Università 16, 35020 Legnaro (PD), Italy; rossella.tessari@unipd.it (R.T.); massimo.morgante@unipd.it (M.M.); matteo.gianesella@unipd.it (M.G.); tamara.badon@unipd.it (T.B.); silvia.bedin@unipd.it (S.B.); elisa.mazzotta@unipd.it (E.M.); michele.berlanda@unipd.it (M.B.)

**Keywords:** transition period, β-hydroxybutyrate, fatty acids, lipid classes, thin layer chromatography

## Abstract

**Simple Summary:**

Dairy cows with a high milk production require an excessive amount of energy in the first phase of lactation. Different metabolic processes are involved to reach the high energy requirement, in particular the mobilization of the adipose tissue which could lead to the development of metabolic diseases, such as subclinical ketosis. Early detection of excessive lipid mobilization could improve the health status of animals and reduce economic losses in farms. The aim of this research is to identify the plasma fatty acids in four lipid classes in order to understand energy metabolism at different levels of ketonemia in dairy cows using Thin Layer Chromatography and Gas Chromatographic techniques (TLC-GC). The research establishes the concentrations of fatty acids, belonging to four plasma lipid classes, in animals with different degrees of hyperketonemia.

**Abstract:**

Excessive mobilization of adipose tissue in high milk producing dairy cows predisposes to metabolic diseases. The aim of this research was to identify the plasma fatty acids in four lipid classes as biomarkers for the diagnosis of hyperketonemia in bovines using thin layer chromatography and gas chromatographic techniques (TLC-GC). Sixty multiparous Holstein–Friesian dairy cows were enrolled in the study. Blood samples from the coccygeal vein were collected and β-hydroxybutyrate (BHB) was evaluated. All animals were divided into three groups on the basis of ketonemia: BHB < 0.50 mmol/L, 0.50 < BHB < 1.0 mmol/L, and BHB > 1.0 mmol/L. Plasma fatty acid concentrations were evaluated in four lipid classes: Free Fatty Acids (FFA), Triglycerides (TG), Cholesterol Esters (CE) And Phospholipids (PL). The concentration of fatty acids was analyzed using TLC-GC. The results showed the following significance in the lipid classes: 19 fatty acids were significant (*p* < 0.053) in FFA, nine fatty acids were significant (*p* < 0.050) in TG, eight fatty acids were significant (*p* < 0.050) in CE and three fatty acids were significant (*p* < 0.049) in PL. Eleven parameters were considered as predictive fatty acids related to animals in hyperketonemia. The FFA increased simultaneously with blood BHB levels, although the identified predictive fatty acids related to the TG and CE lipid classes decreased, meanwhile the BHB values increased. In the PL lipid class, no fatty acids were predictive.

## 1. Introduction

In the transition period, the strong metabolic, physiological, and nutritional changes due to the continuous energy demand from the fetus, lactogenesis, and the reduced Dry Matter Intake (DMI) results in an energy deficiency [1]. This energy disequilibrium is expressed through a negative energy balance (NEB) [2].

Dairy cows provide energy through a metabolic pathway called ketogenesis. An excessive formation of ketone bodies such as acetoacetate (AcA), acetone (Ac) and β-hydroxybutyrate (BHB) can result in a metabolic disorder, nominated ketosis [3]. Ketosis can be classified into the subclinical or clinical. Subclinical ketosis is characterized by an increase in the concentrations of ketone bodies in the blood, urine, or milk without the presence of clinical signs [4,5]. The threshold value of the blood BHB concentrations used to diagnose subclinical ketosis have a range of value from 1.0 mmol/L to 1.4 mmol/L [6]. Clinical ketosis is characterized by visible clinical signs such as a decreased appetite, weight loss, and reduced milk production. A blood BHB concentration above 2.9 mmol/L is the threshold of clinical disease [7,8].

In early lactation, the metabolism becomes purely catabolic through the mobilization of adipose tissue [9]. The NEB leads to a release of free fatty acids into the blood as a result of an enhancement of lipolysis and a decrease in lipogenesis [10]. 

The fatty acids present in the blood circulation are divided into various lipid classes (or fractions) that include the Phospholipids (PL), Neutral Lipids (NL), and Non-Esterified Fatty Acids (NEFA). The NL fraction consists of Monoglycerides (MG), Diglycerides (DG), Triglycerides (TG), and Cholesterol Esters (CE). The lipids belonging to the NL fraction and the PL are transported in circulation by transport lipoproteins. NEFAs are transported by serum albumin and could be used directly as an energy source by various tissues or be taken up by the liver [11,12]. The 15%–20% of the total NEFA present in the blood circulation is derived from the liver and undergoes three different processes [12,13]. For the dairy cow in an energy deficit, the main process is the completely oxidized of NEFA to carbon dioxide to provide energy for the liver through the tricarboxylic acid cycle (TCA) [13].

Furthermore, in an extreme negative energy balance, the NEFA can be re-esterified in triglycerides and deposited in the liver or be transformed into ketone bodies such as β-hydroxybutyrate when the enhanced gluconeogenetic process subtracts the oxalacetate to the tricarboxylic acid cycle [8,13,14]. In this way, bovines with a high mobilization of adipose tissue can be predisposed to diseases such as hepatic lipidosis and subclinical-clinical ketosis [15,16]. In the transition period, the lipolysis induced by NEB involves the modification of the plasma concentration of fatty acids. Consequently, the quantity of total fatty acids in early lactation is higher than the quantity of total fatty acids presents in medium-late lactation and in the dry period [17]. The fatty acids that most diversify their composition are long chain fatty acids (LCFA) and, in particular, polyunsaturated fatty acids (PUFA) belonging to the class of Omega 6 [17,18]. 

The identification of new biomarkers for the early diagnosis of subclinical–clinical ketosis was the basis of our research. NEFA and BHB are currently the biomarkers used for the diagnosis of excessive lipid mobilization and hyperketonemia in bovine peripartum [7,19]. Elevated blood concentrations of these two parameters could be considered as risk indicators of developing diseases such displaced abomasum, clinical ketosis, metritis, and retained placenta [9]. An evaluation of their concentrations allowed for the implementation of nutritional and management strategies in order to avoid the development of disease [20]. 

Recently, new methods have been used to highlight clinical parameters to deepen the knowledge of the metabolism or metabolomics of human and animal bodies. Through the study of metabolites, it is possible to observe changes in the profile of the chemical and biochemical composition of biological fluids and tissues and therefore identify the alteration of the normal physiology of the organism. Thin layer chromatography and gas chromatographic techniques (TLC-GC), one of the most prevalent technologies of metabolomics, have allowed for the preparation and purification of molecules from the biological matrix as fatty acids from blood samples [21].

Currently, the threshold for the diagnosis of subclinical and clinical ketosis is bibliographically available [12]. The aim of the study was to investigate the association between serum concentrations of BHB and the changes in the fatty acid concentration of the plasma lipid fraction in a population of Holstein–Friesian dairy cows in early lactation using TLC-GC. The identification of serum markers of hyperketonemia would be useful to prevent medical treatments and to reduce the economic losses deriving from subclinical-clinical ketosis. 

## 2. Materials and Methods 

### 2.1. Animals

Sixty Holstein–Friesian dairy cows were enrolled from a single high-producing dairy farm in Padua, Italy (45°36’ N, 11°40’ E, 23m above sea-level). The farm had 600 bovines, 400 animals in milk with a preceding production of 9693 ± 235 L. All animals had a dry period of 55 ± 5 days in the farm. Total Mixed Ration (TMR) and the chemical composition of lactation diet was reported in Table 1. Water was available ad libitum. The group of animals enrolled were in early postpartum, with an average of 26.5 ± 1.5 days in milk (DIM). 

All animals were evaluated by the Veterinarian of University of Padua (Italy) and were clinically healthy. The dairy cows with clinical diseases (metrits, retained placenta, abomasal displacement, milk fever, etc.) were excluded from the research.

### 2.2. Experimental Design

Blood sampling was carried out late in the morning using coccygeal vein puncture. The BHB was measured using the Nuova Biomedical Express (Nuova Biomedical, Runcorn, UK) digital reader before collecting the blood sample. All the blood samples were analyzed using the BHB strips test (Stat Strip Ket, Nova Biomedical, Reggio Emilia, Italy).

Three blood samples were collected with the vacutainer system for each enrolled cow. The first aliquot was taken using a tube containing EDTA (5 mL; Terumo Venoject, Leuven, Belgium) to avoid the activation of the coagulation cascade while the other two aliquots were stored in Venosafe tubes containing Clot Activator (9mL; Terumo Venosafe, Leuvel, Belgium). The EDTA samples were centrifuged at 1500 g for 10 min using a centrifuge (LW Scientific Ultra 8V 800-726-7345, Lawrenceville, Georgia) in the farm. For each sample with EDTA, 250 µL of plasma was taken with a micropipette (Pipetman Classic P1000) and introduced into a 1.5 ml capacity Eppendorfs containing 5 mg of pyrogallol. The samples were stirred until the pyrogallol was completely dissolved. The effect of pyrogallol on plasma was to prevent the oxidation of unsaturated fatty acids. The samples were stored at −80 °C until the analysis.

The Eppendorfs and the Clot Activator tubes were refrigerated at 4 °C and transported to the laboratory of the Department of Animal Medicine, Production and Health (MAPS) of the University of Padua (Italy). The blood samples containing Clot Activator were centrifuged in the laboratory (Heraeus Labofouge 400, Thermo Scientific, Milan, Italy). The serum extracted was stored at −20 °C for subsequent biochemical analysis.

### 2.3. Blood Analysis

Serum biochemistry and plasma gas chromatography (GC) were performed in the laboratory. The serum was assessed employing automatic clinical chemistry analyzer (BT3500 Biotecnica Instrument SPA, Rome, Italy). Serum BHB concentrations were measured using β-hydroxybutyrate Enzymatic Kinetics (Randox, Milan, Italy; BHB, mmol/L). 

On the basis of serum BHB concentrations obtained in the laboratory, all animals were divided into three different groups: Group BHB 0, healthy animals with lower BHB value (BHB < 0.50 mmol/L; 17 animals); Group BHB 1, healthy animals with medium serum BHB value (0.50 ≤ BHB < 1.0 mmol/L; 22 animals); and Group BHB 2, sick animals with higher serum BHB values (BHB ≥ 1.0 mmol/L; 21 animals). 

The healthy animals (BHB < 1.00 mmol/L,) were divided into two groups to evaluate the different concentrations of plasma fatty acids in healthy dairy cows and no references in the literature are recorded on the quantification of fatty acids.

In order to perform the GC, the samples were exposed to three sequential tests: extraction of lipids from the plasma, separation of the lipid classes by Thin Layer Chromatography (TLC), and methylation of the carbon chain. The method used for the quantification of plasmatic fatty acids is in accordance with the study of Carnielli et al. [22]. Before starting these procedures, the samples were mixed with the internal standards (C9–C15 or C17) of every lipid class, for the quantification of the single fatty acids. 

The first process allowed the extraction of plasma lipids (organic substances) from the aqueous component. This biphasic separation occurred using a mixture of chloroform and methanol (2:1, v/v), as described by Folch et al. [23]. This extraction allowed the formation of two distinct phases: the supernatant, containing methanol and H_2_O, and the subnatant, containing chloroform and lipids, interrupted by a thin protein layer [24]. The solution was enriched with a NaCl saturated solution to intensify the separation between the aqueous component and the lipid matrix. The organic component was subsequently exposed to the heating block at 37 °C and to a slight flow of nitrogen, in order to allow the evaporation of the organic solvent in which the lipids were dissolved and to avoid the oxidation of unsaturated fatty acids.

Afterward, the TLC was performed. The samples were dissolved in 100 µl of chloroform and BHT 50 mg/L as antioxidant and lay on the deposition line of the TLC. The separation of lipid classes was generated due to a different degree of affinity between the compounds present in the sample, the stationary phase, and the mobile phase as described by Santiago and Strobel [25]. Fluorescein was nebulized in the chromatographic plate which generated a chromatic contrast. At the end of the procedures, four different compounds were obtained for each sample: FFA (Free Fatty Acids), TG (Triglycerides), CE (Cholesterol Esters), and PL (Phospholipids).

The third process involved the methylation of the lipid classes using 3N methanolic hydrochloric acid. The samples obtained were placed in an oven for one hour at 100 °C and then neutralized with a solution of potassium carbonate (K_2_CO_3_). 

Finally, for each plasma sample 37 fatty acids were obtained per lipid classes. The fatty acid methyl esters were separated and quantified in splitless mode by GC using a TRACE GC/MS (Thermo Quest, Milan, Italy) equipped with a flame ionization detector (FID) and a polar fused-silica capillary column (Capillary Column Omegawax, 30 m × 0.25 mm × 0.2 µm film). Helium was used as the carrier gas at a flow rate of 1 ml/min. Data for plasma fatty acid were calculated in mg/dL.

### 2.4. Statistical Analysis

Serum and plasma fatty acid methyl ester data were analyzed using the SAS system software (version 9.4; SAS Institute Inc., Cary, NC, USA). The General Linear Model (GLM) analysis was performed for repeated measurements in order to evaluate the differences in the composition of plasma fatty acids in the four lipid fractions. The Bonferroni’s test was achieved to analyze interparametric differences within the three classes. 

The decision algorithm of Boruta test (R2 software, Santa Monica, CA) was used to select the fatty acids with a predictive function in order to diagnose different state of ketonemia in relation to fatty acids.

The Receiver Operating Characteristic (ROC) (MedCalc Sofware Ltd, Ostend, Belgium) curves were performed to establish the threshold value of each predictive fatty acid, beyond which the animals were considered ketonemic. The ROC curves were derived from the analysis of plasma fatty acids data of all experimental animals (BHB0-BHB1-BHB2) with the discriminant of sick animals (BHB2). The ROC curve established the cut-off of each fatty acid for the diagnosis of hyperketonemia. Therefore, all possible values were analyzed through the diagnostic test and the ROC curve represents the proportion between sensitivity (SE: true positives) and 1-specificity (SP: false positives). Compared to the random classification line, the highest point of the curve was the threshold value. The ROC curve established a cut-off for the fatty acids selected by the Boruta decisional algorithm. The area under the curve (AUC) expresses the diagnostic power of the test [26].

## 3. Results

### 3.1. Descriptive Statistics

Table 2 shows the mean values (±SEM) regarding BHB, DIM, Body Condition Score (BCS), parity and yield milk produced (Kg/day) for all enrolled animals, based on the three classes of BHB (BHB0, BHB1, and BHB2). The mean values were respectively 0.42 mmol/L for BHB0 group, 0.71 mmol/L for BHB1 group, and 1.36 mmol/L for BHB 2 group.

Afterwards, it was possible to evaluate the different composition of fatty acids in the four different lipid classes of FFA, TG, CE, and PL through the analyses performed in TLC-GC. The mean values (± SEM) of the four lipid fractions were described in Table 3. The composition of the different fatty acids related to these four lipid classes (FFA, TG, CE, PL) was compared to changes in blood concentrations of β-hydroxybutyrate (BHB0 vs BHB1 vs BHB2).

### 3.2. Effect of Blood BHB on Lipid Class of Free Fatty Acids

Table 4 represents the mean values (± SEM) of the different plasma fatty acid related to the lipid class of free fatty acid, comparing the values in the three BHB groups. C14:1ω5 (*p* = 0.002), C16:1ω7 (*p* = 0.038), C18:0 (*p* = 0.012), C18:1ω9 (*p* = 0.024), C18:1ω7 (*p* = 0.020), C18:2ω6 (*p* = 0.001), C18:3ω6 (*p* = 0.050), C18:3ω3 (*p* = 0.002), C18:4ω3 (*p* = 0.007), C20:1ω9 (p = 0.020), C20:1ω7 (*p* = 0.003), C20:2ω6 (*p* = 0.024), C20:3ω6 (*p* = 0.028), C20:4ω3 (*p* = 0.018), C20:4ω6 (*p* = 0.001), C22:0 (*p* = 0.020) and C22:6ω3 (0.034 mg/dL) were the significant fatty acids in the lipid class of FFA (*p* < 0.05). Furthermore, there is a significant difference in total fatty acids (*p* = 0.053) expressed in mg/dL.

### 3.3. Effect of Blood BHB on Lipid Class of Triglycerides

Table 5 reports the mean of the different plasma fatty acids (± SEM) related to the lipid class of TG, comparing the values in the three BHB groups. C14:0 (*p* = 0.050), C14:1ω5 (*p* = 0.040), C17:0 (*p* = 0.001), C18:0 (*p* = 0.042), C18:1ω7 (*p* = 0.050), C20:3ω3 (*p* = 0.038), C20:4ω3 (*p* = 0.035), C20:4ω6 (*p* = 0.013) and C22:1ω9 (*p* = 0.018) were the significant fatty acids in the lipid class of TG (*p* < 0.05).

### 3.4. Effect of Blood BHB on Lipid Class of Cholesterol Esters

Table 6 shows the mean values (± SEM) of the different plasma fatty acids related to the lipid class of CE, comparing the values in the three BHB groups. C12:0 (*p* = 0.050), C18:3ω3 (*p* = 0.035), C20:4ω6 (*p* = 0.047), C20:3ω3 (*p* = 0.050), C20:5ω3 (*p* = 0.040), C22:2ω6 (*p* = 0.032), C22:3ω6 (*p* = 0.040) and C23:0 (*p* = 0.010) were the significant fatty acids in the lipid class of CE (*p* < 0.05).

### 3.5. Effect of Blood BHB on Lipid Class of Phospholipids

Table 7 reports the mean (± SEM) of the different plasma fatty acids related to the lipid class of phospholipids, comparing the values in the three BHB groups. C22:1ω9 (*p* = 0.018), C22:2ω6 (*p* = 0.0482), and C16 DMA (*p* = 0.0491) were the significant fatty acids in the lipid class of PL (*p* < 0.05).

### 3.6. Predictive Fatty Acids and Cut-Off Related to Animals in Hyperketonemia

**Figure 1:**
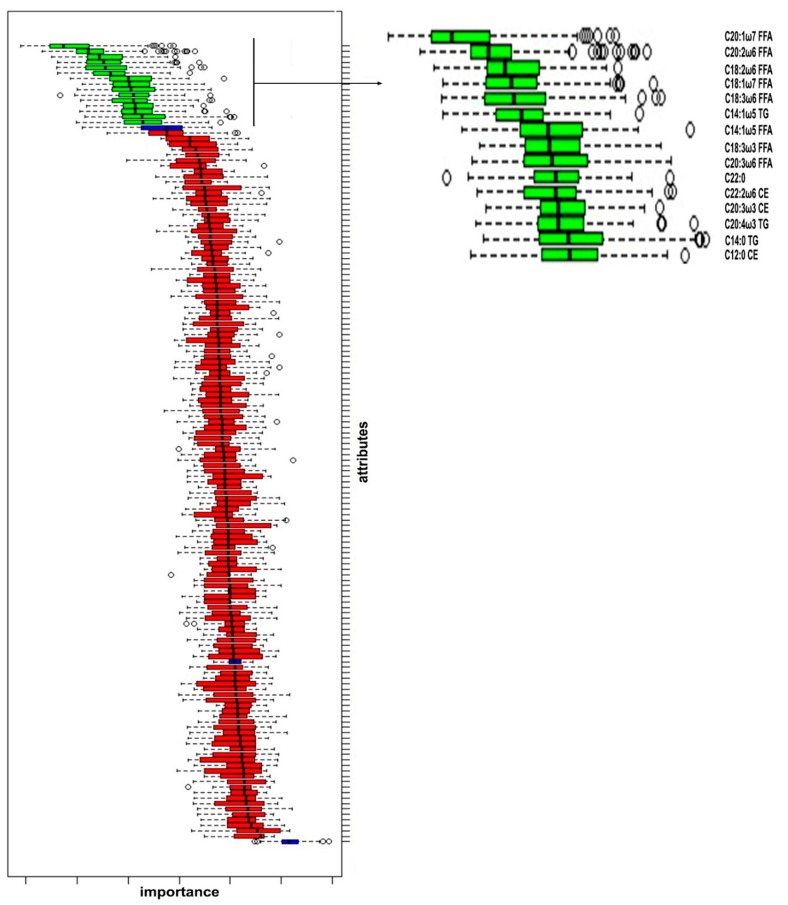
Box plot of Boruta decisional algorithm and enlargement of the green box plot with nomenclature of the main predictive fatty acids. A color code was used to facilitated the reading of the graph. The green box plot represented the fatty acid with the greatest predictive function while the fatty acids with null predictive function were represented by the red box plot.

The fatty acid with the predictive function was identified through the Boruta decisional algorithm. The algorithm selected 15 significant fatty acids and these were represented in the box Figure 1. 

According to the results, the selected plasma fatty acids were reported in four lipid classes related to their predictive function respectively:

- Eight predictive fatty acids belonging to the fraction of FFA (C20:1ω7, C20:2ω6, C18:2ω6, C18:3ω6, C14:1ω5, C18:3ω3, C20:3ω6, C22:0);
- Four predictive fatty acids deriving from the lipid class of TG (C18:1ω7, C14:1ω5, C20:4ω3, C14:0);
- Three predictive fatty acids deriving from the lipid class of CE (C22:2ω6, C20:3ω3, C12:0);
- No fatty acids belonging to the lipid class of PL were found.

The predictive fatty acids for the algorithm are listed in Table 8 with relative average, median, minimum value, maximum value, and normal values. Once more, the fatty acids are reported according to decreasing predictive function. 

The analysis of the ROC curve was performed on the 15 predictive fatty acids (green box diagram of Figure 1). The ROC curves were derived from the analysis of plasma data of all experimental animals (BHB0- BHB1- BHB2), discriminating with BHB values of the sick animals (BHB2). The ROC curve established the cut-off of each acid for the diagnosis of hyperketonemia. The interpretation of this critical threshold depends on the AUC. 

**Figure 2:**
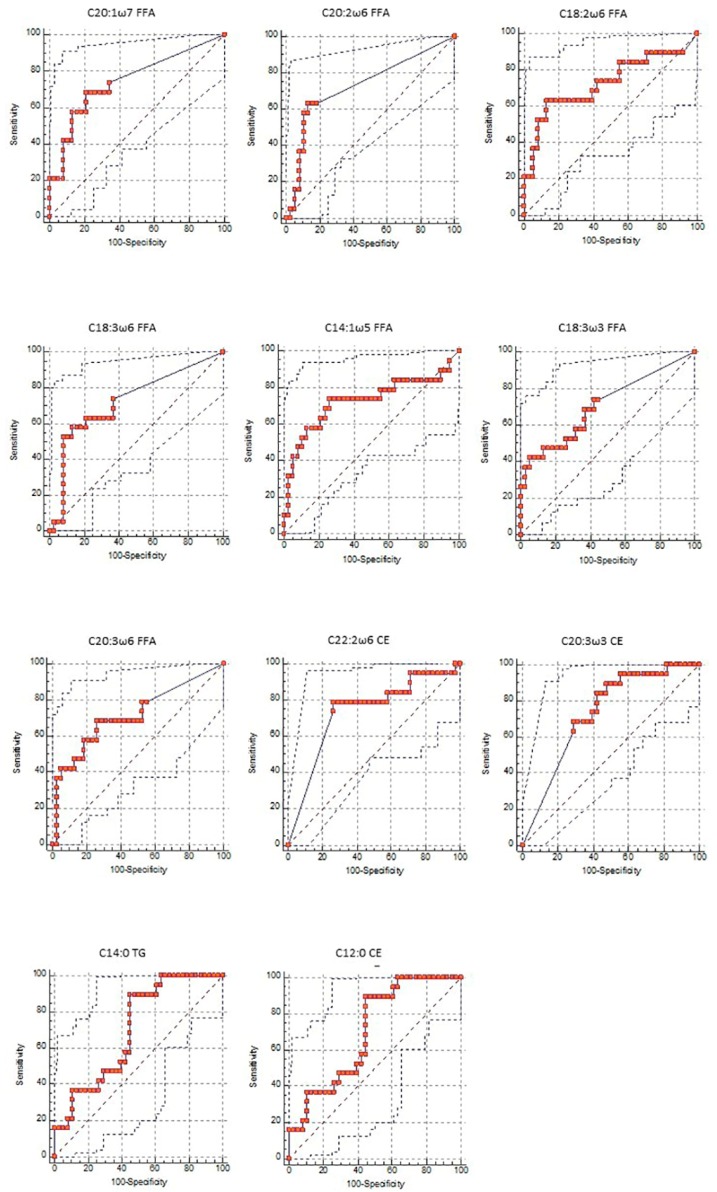
Receiver Operating Characteristic (ROC) curve of predictive fatty acids having Area Under the Curve (AUC) greater than 0.70 and listed with decreasing predictive function obtained from Boruta decisional algorithm.

Table 9 reports the predictive plasma fatty acids derived from the Boruta decisional algorithm with the ROC curve analysis. If the AUC is greater than 0.70 the test performed for plasma fatty acid is considered moderately predictive. We selected 11 significant fatty acids, with AUC greater than 70%, whose ROC indicates a threshold value with a moderately accurate diagnostic function and we eliminated 4 fatty acids because the AUC was lower than 0.70 (C18:1ω7 TG, AUC = 0.618; C14:1ω5 TG, AUC = 0.514; C22:0 FFA AUC = 0.634; C20:4ω3 TG, AUC = 0.637). In Figure 2 were reported the ROC curve of the fatty acid with diagnostic function.

## 4. Discussion

In this study, results showed that the DIM, BCS, parity, and yield milk did not generate significant differences. 

Dairy cows, with loss of one BCS point in the transition period, are more predisposed to develop metabolic pathologies [15]. In our study, repeated measures of BCS were not evaluated to calculate the loss of nutritional status and a significant difference was not generated.

NEFA and BHB are markers of a NEB condiction; in our study, the plasma fatty acids showed significant differences related to the variation of BHB in the blood (*p* = 0.0001) according to the available literature (Table 2) [27]. 

We investigated the concentrations of the different plasma fatty acids of the four lipid classes in function of three different blood BHB concentrations. 

Regarding the different four lipid fractions (FFA, TG, CE, and PL; respectively from Table 4 to Table 7), the values of some fatty acids change according to the ketonemia. In the study of Yamdagni and Schultz [28], ketonemic animals presented an alteration in the concentrations of plasma lipid fractions compared to cows with normal blood BHB. The concentrations of TG, CE, and PL decremented respectively to the 53%, 34%, and 38%, when compared to the concentrations of bovines in healthy status [28]. Instead, the lipid class of FFA had tenfold higher concentrations in ketotic cows [28].

In the study of Brumby et al., fasting animals had decreased serum concentrations of PL and CE but exhibited increased concentrations of FFA. The fasting period imposed to induce ketosis on the animals of that study can be compared to the reduction of the DMI that occurs in the dairy cows of our study [29]. According to the mentioned studies, our results report that the concentration for the lipid classes of TG, CE, and P were lower in the BHB 0 and BHB 1 group and the FFA lipid class had higher concentrations in the group with BHB> 1.0 mmol/L (hyperketonemic group).

### 4.1. Effect of Blood BHB on Lipid Class of Free Fatty Acids

Several studies observed that the hydrolysis of triglycerides deposited in adipose tissue is one of the most considerable catabolic process activated to obtain energy in bovines with high milk production [13,30]. This process generates an increase in plasma FFA positively correlated with the increase in NEB [1,31].

Palmitic acid (C16:0), palmitoleic acid (C16:1), stearic acid (C18:0), and oleic acid (C18:1) are the main fatty acids present in the adipose tissue [32].

Puppel et al. found that NEB induces the mobilization of long chain fatty acids (LCFA) from adipose tissue specifically C16:0, C18:0 and C18:1 [33]. According to Rukkwamsuk et al., the plasma concentrations of C18:2 also increase postpartum as a result of an increase in lipolysis of the adipose tissue [32].

In our research, we found a significant increase in the plasma concentrations of C16, C18, C18:1, and C18:2 in the group BHB2 compared to cows with BHB blood concentrations lower than 1.0 mmol/L (BHB0 and BHB1). These results confirmed that an excessive increase in lipid mobilization develops a hyperketonemic state. NEB associated with a mobilization of adipose tissue was observed through the evaluation of the amount of total fatty acids in ketotic cows (11.680 mg/dL) compared to healthy cows (7.694 mg/dL) with a lower NEB. 

As previously reported, seventeen of nineteen significant fatty acids in the FFA lipid class (Table 4) presented a higher concentration in groups of animals with BHB > 1.0 mmol/L. 

In many studies, the threshold concentration of blood BHB used to diagnose subclinical ketosis is higher than 1.2 mmol/L [20,27]; this justifies the greater presence of mobilized fatty acids in the enrolled bovines in BHB 2 group.

We observed that the amount of fatty acids did not present a concentration directly proportional to the BHB blood values. The bovines with medium BHB values (0.5mmol/L–1.0 mmol/L) showed the lowest concentrations of fatty acids compared to the group of animals with BHB < 0.5mmol/L. In current literature, there are no studies which compare the concentrations of plasma fatty acids in group of dairy cows with mean BHB values lower than 1.0 mmol/L. Further studies should be performed to evaluate the ideal BHB in which, lipid mobilization would not cause metabolic diseases. Our results may exclusively hypothesize that concentrations of BHB higher than 0.5 mmol/L and lower than 1.0 mmol/L would generate a lower degree of mobilization of the adipose tissue.

### 4.2. Effect of Blood BHB on Lipid Class of Triglycerides

Plasma TG concentrations in the transition period increase during the postpartum period. In the study by Fiore et al., it is reported that the TG amount in plasma was higher around the 30th day of DIM compared to the 10th day of DIM [34]. It is important to highlight that the study was carried out in cows which did not exceed the cut-off of normal ketonemia [34]. This study was based on a cross sectional analysis in which the parameters were assessed in a single time point, therefore we could not compare the parameters at different lactation days (26.5 ± 1.5 DIM), but it was possible to evaluate the plasma concentrations of TG that decreased in healthy cows (BHB < 1.0mmol/L) to cows in hyperketonemia. 

The decrease in plasma TG concentrations in BHB 2 group could be considered as an accumulation of lipids in the liver. The condition of hepatic infarction negatively influences the function and the synthesis of Very Low Density Lipoprotein (VLDL) in this organ and consequent decrease of TG concentrations in blood [29,35]. Oikawa et al. [36] showed that the ratio between blood VLDL, the main carrier of TG, and plasma NEFA increases at the beginning of lipid mobilization (first day after calving) and decreases in the period of maximum NEB/fasting [36]. Furthermore, TG concentrations in blood are lower in bovine developing fatty liver after parturition [37]. In fact, during the first four weeks of lactation, the liver has a lipid uptake that exceeds the capacity of oxidation or secretion leading to disorders [38]. As expected, in our research study, the total of the fatty acids from the lipid class of triglycerides was equal to 7.536 mg/dL in sick cows (maximum NEB) compared to a considerable amount present in healthy cows, 10.999 mg/dL (lower NEB). Five out of nine significant plasmatic fatty acids of this lipid class tended to decrease with the increases of BHB concentration and therefore greater lipomobilization (Table 5). 

### 4.3. Effect of Blood BHB on Lipid Class of Cholesterol Esters 

The fatty acids deriving from the lipid fractions of CE also decreased as well as the blood BHB increased. In our study, the low concentration of fatty acids is shown through the evaluation of the amount of total fatty acids in ketotic cows (164.219 mg/dL, BHB 2) with more NEB, compared to healthy cows with low or high blood BHB concentrations (170.815 mg/dL, BHB 0; 213.387 mg/dL, BHB 1). 

The ruminants show a decrease of this lipid class after parturition caused by the decrease of the enzyme lecithin cholesterol acyl-transferase (LCAT) [35,39].

LCAT is a serum enzyme and is synthesized in the liver. The reaction catalyzed by LCAT consists of the transfer of fatty acids from lecithin to cholesterol consequently producing esterified cholesterol (CE). The substrate of LCAT is the free cholesterol present in high-density lipoproteins (HDL) and the products of the reaction are lipoproteins rich in cholesterol esters [39].

This enzyme decreases before and immediately after parturition in bovines that have an excess of hepatic lipidosis caused by an excess of NEB [35,39]. The reduction in enzymatic activity of LCAT is caused by fat infiltrations in the liver and this decreased activity explains the low level of cholesterol esters [40]. Moreover, in Nakagawa and Katoh’s study, the dairy cows that presented a greater decrease in the LCAT enzyme were those in ketosis [40]. Furthermore, dairy cows in nutrition-induced ketosis present an alteration of the signaling and downregulation of liver genes that predispose to the reduction of the synthesis of CE [41]. In summary, the excessive liver lipidosis and downregulation of liver genes predispose to a reduction in the activity of LCAT which is expressed as a decrease in CE [40,41].

As previously mentioned, in our study, seven out of eight significant fatty acids in the CE lipid class (Table 6), presented a lower concentration in BHB 2. Conversely to what we expected, we observed that the concentrations of fatty acids were not indirectly proportional to the blood BHB values. From our results, the group of cows with intermediate BHB value (0.5 mmol/L–1.0 mmol/L) showed the higher fatty acid concentrations. Once again, we may assume that BHB concentrations higher than 0.5 mmol/L and lower than 1.0 mmol/L lead to better hepatic function, improving CE production (BHB 1), although no specific comparable values for the concentration of fatty acids are reported in current literature.

### 4.4. Effect of Blood BHB on Lipid Class of Phospholipids

In this study, plasma fatty acids from the lipid fractions of PL tended to decrease as blood BHB increased. The lower concentration of fatty acids was proved through the evaluation of the amount of total fatty acids in ketotic cows (81.490 mg/dL, BHB 2) compared to animals with BHB values lower than 0.5 mmol/L (84.307 mg/dL, BHB 0).

According to Van den Top et al., phospholipid transfer proteins (PLTP) have a positive correlation with LCAT; in fact, in their study, they observed a decrease of both lipoproteins immediately postpartum [35]. For this reason, we can assume that the decrease of fatty acids derived from the lipid class of cholesterol esters is positively correlated with the decrease of the fatty acids from the lipid class of phospholipids. No bibliographical references described a decrease of PLTP enzymes in hyperketonemic bovines, but the previous study reported a sudden decrease in phospholipids in transport lipoproteins (VLDL, IDL) in the first few weeks after calving [35]. 

In the study of Nakagawa and Katoh, ketotic bovines showed a decrease in the concentrations of PL and CE associated with a decrease in the LCAT enzyme during early lactation contrary to cows with normal BHB and NEFA values [40]. Moreover, in our research, bovines with concentrations of β-hydroxybutyrate greater than 1.0 mmol/L (BHB 2) showed a reduction of fatty acid belonging to CE and PL classes contrary to bovines belonging to the group with normal BHB value (<1.0 mmol/L) (Table 7).

Gross et al. conducted a study where the variation of lipid metabolism was evaluated in bovines with a NEB at different stages of lactation (DIM 0 e DIM 100) [42]. From the study, it emerged that the PL and cholesterol concentrations tend to decrease in immediately post calving cows with physiological NEB compared to fasting cows at 100 DIM [42]. In this study, the concentrations of LCAT and PLTP were not directly proportional [42]. Further studies related to enzymatic metabolomics would be needed to correlate the mobilization of plasma fatty acids and to identify which enzymes are involved in the synthesis of molecules. 

### 4.5. Fatty Acid Cut-Off Related to Hyperketonemia

The Boruta decisional algorithm allowed us to select 15 predictive fatty acids: eight predictive fatty acids were into the lipid class of FFA, and as previously reported in literature, they tended to enhance as lipid mobilization increased [1]; three CE fatty acids decreased, as well as the predictive fatty acids; four TG fatty acids showed an anomalous trend when compared to literature. In fact, as reported in the study by Oikawa and Oetzel, C14:0 and C18:1ω7 (TG) decreased, but C14:1ω5 and C20:4ω3 (TG) tended to increase, in contrast with literature [43]. In this class of lipid, ROC curves do not confirm three fatty acids (C18:1ω7, C14:1ω5, and C20:4ω3). In fact, these parameters showed an AUC of less than 70%. Therefore, C18:1ω7, C14:1ω5, and C20:4ω3 extrapolated from the Boruta algorithm were considered as non-accurate diagnostic parameters by the ROC analysis. Furthermore, C14:1ω5 and C20:4ω3 presented an increase in the concentrations in response to the increase in blood BHB. The increase of these two fatty acids, which was not diagnostic in our ROC analysis for subclinical ketosis detection, was reported to have a similar tendency by other authors [28]. In the transition period, the plasma triglycerides tend to decrease their plasmatic concentration as the hepatic lipidosis increases [36,44].

In our study, one saturated fatty acid (C22:0 FFA) predictive for the Boruta decisional algorithm was not accurate according to the ROC analysis. Specifically, C22:0 increased in plasma concentrations from healthy (0.007 mg/dL) to ketonemic bovines (0.020 mg/dl); however, in literature, it is reported that saturated fatty acids tend to decrease after calving when the NEB is greater [17]. Regarding C22:0, the AUC was lower than 70% and excluded this fatty acid as a possible marker of hyperketonemia. This result was supported by literature [17].

Therefore, we examined the different chemical compositions of the fatty acids, evaluating the presence of double bounds and their position in respect to the terminal methyl group. 

Predictive values include five polyunsaturated fatty acids belonging to the family of omega 6 (PUFAω6); two monounsaturated fatty acids (MUFA); two polyunsaturated fatty acids belonging to the family of omega 3 (PUFA ω3); and two saturated fatty acids (SFA). 

In the study of Abdel-Raheem et al. [17], fatty acids with different chemical compositions are mobilized according to the lactation phase. In fact, the PUFA omega 6 increased in mid lactation, and the MUFA had higher concentrations in the first lactation phase where the BEN and lipomobilization had maximum expression. The concentrations of PUFA omega 3 decreased in the early lactation phase, and the SFA had lower values in all lactation periods compared to the dry period [17]. Our results showed that an exacerbated state of NEB, resulting in hyperketonemic state, could increase the lipid mobilization with the same chemical composition of the fatty acids specifically mobilized in early–medium lactation: MUFA and PUFA Omega 6. This data supports the hypothesis that the lipid mobilization of fatty acids, belonging to a given chemical composition, might be related to both the lactation stage and the blood BHB. Further studies would be needed to evaluate the correlation between the chemical compositions of the mobilized fatty acids and the blood BHB independent of the lactation phase.

In our study, the values of the most significant parameters for the Boruta decisional algorithm with AUC > 0.70 (Table 9) were, respectively, two MUFA, C20:1ω7 (FFA, AUC = 0.746) and C14:1ω5 (FFA, AUC = 0,724); and three PUFA ω6 C20:2ω6 (FFA, AUC = 0.729), C18:2ω6 (PUFA ω6, FFA, AUC = 0.726), and C18:3ω6 (FFA, AUC = 0.724). These parameters proved an increase in the plasmatic amount of these fatty acids from healthy dairy cows (respectively 0.007 mg/dL, 0.061 mg/dL, 0.078 mg/dL, 0.361 mg/dL and 0.009 mg/dL) to animals in hyperketonemia (respectively 0.029 mg/dL, 0.099 mg/dL, 0.132 mg/dL, 0.511 mg/dL and 0.022 mg/dL). This indicates an enhancement in lipolysis with the increase of NEB as in the study mentioned above [17]. Observing the predictive saturated fatty acids that had an AUC > 70%, such as C12:0 (CE, AUC = 0.738) and C14:0 (TG, AUC = 0.701), it was evident that there was a decrease in plasmatic concentration from healthy (3.133 mg/dL and 0.409 mg/dL) to hyperketonemic cows (1.736 mg/dL and 0.167 mg/dL). Furthermore, the significant PUFA ω3, C20:3ω3 (CE, AUC = 0.731) showed a decreasing trend from healthy (0.066 mg/dL) to sick dairy cows (0.026 mg/dL). 

Only two fatty acids with an AUC greater than 0.70 had an anomalous trend related to their chemical composition, while they showed a normal trend compared with their constituent lipid classes. 

The fatty acid C18:3ω3 (PUFAω3, AUC = 0.706), referring to the lipid class of FFA, tended to increase as the β-hydroxybutyrate increases, conversely C22:2ω6 (PUFAω6, AUC = 0.733), belonging to the lipid class of cholesterol esters, tended to decrease. The threshold value indicates the concentrations of fatty acids required for the diagnosis of hyperketonemia. The cut-off is used to make a diagnosis based on a scientifically established numerical value.

In the hyperketonemic status, some fatty acids increase their plasma concentrations above the threshold limit while other fatty acids values decrease below the limit. This depends on the fatty acid selected as a diagnostic parameter.

## 5. Conclusions

In our study, the trend of plasma fatty acids in four lipid classes (FFA, TG, CE and PL) with different blood concentrations of β-hydroxybutyrate were comprehensively described and 11 predictive parameters were identified. The change in the composition of plasma fatty acids in the first lactation phase confirmed the applicability of TLC-GC as a predictive technique in the diagnosis of hyperketonemia. 

The evaluation of the four different plasma lipid classes showed that FFA and CE were the lipid fractions with the greatest predictive function. The fatty acids belonging to these lipid classes showed a greater variation in plasma quantities as the concentrations of ketone bodies increased. 

Finally, we concluded that the main fatty acid of the FFA class with high predictive power for the diagnosis of hyperketonaemia was C20:1ω7 (*p* = 0.746), whose plasma concentration value was greater than 0.0058 mg/dL, having diagnostic function. The main fatty acid with diagnostic function belonging to the CE was C12:0 (*p* = 0.738) whose concentration equal to or less than 0.226 mg/dL had a discriminating function between healthy animals and animals with hyperketonaemia.

Future studies will be necessary to evaluate the variation of plasma fatty acids in the last phase of gestation to make an early diagnosis and improve the treatment of dairy cows with hyperketonemia. 

## Figures and Tables

**Table 1:** Chemical composition of the Total Mixed Ration (TMR) and feedstuff with % dry matter (DM) in lactation period.

Chemical Composition		Feedstuff	Dry Matter (%, DM)
TDMI ^1^ (kg/d)	24.2	Alfalfa haylage	27.38
UFL ^2^	0.96	Alfalfa hay	21.20
CP ^3^ (%, DM)	15.20	Cottonseed meal	7.53
PD ^4^ (%, DM)	12.11		
PDIN ^5^ (%, DM)	11.19	Concentrate mix:	43.89
PDIE ^6^ (%, DM)	11.32	Corn	41.30
PDIA ^7^ (%, DM)	5.49	Barley	19.30
NDF ^8^ (%, DM)	31.35	Wheat	16.40
ADF ^9^ (%, DM)	18.84	Soybean meal	8.00
ADL ^10^ (%, DM)	2.72	Corn gluten meal	8.00
EE ^11^ (%, DM)	4.60	Molasses	3.00
ASH ^12^ (%, DM)	7.35	Sodium bicarbonate	2.85
ST ^13^ (%, DM)	24.60	Vitamin D	0.06
NSC ^14^ (%, DM)	41.37	Vitamin A	0.06
Ca ^15^ (%, DM)	0.80	Vitamin E	0.03
P ^16^ (%, DM)	0.40	Mineral salts	1.00

^1^ Total Dry Matter Intake; ^2^ Unitè Fouragère Lait; ^3^ Crude Protein; ^4^ Protein Digestible; ^5^ Protein Digested In The Small Intestine When Rumen-Fermentable Nitrogen Is Limiting; ^6^ Protein Digested In The Small Intestine When Rumen-Fermentable Energy Is Limiting; ^7^ Dietary Protein Undegraded In The Rumen But Truly Digestible In The Small Intestine; ^8^ Neutral Detergent Fiber; ^9^ Acid Detergent Fiber; ^10^ Acid Detergent Lignin; ^11^ Ether Extract; ^12^ Ashes; ^13^ Starch; ^14^ Non-Structural Carbohydrates; ^15^ Calcium; ^16^ Phosphorus.

**Table 2:** Mean value (±SEM) of β-hydroxybutyrate (BHB), days in milk (DIM), Body Condition Score (BCS), parity, and daily milk yield.

Parameters	Group BHB 0	Group BHB 1	Group BHB 2	SEM	*p*-Value
BHB (mmol/L) ^1^	0.42	0.71	1.36	0.018	0.0001
DIM ^2^	26.90	27.60	24.90	2.987	NS ^3^
BCS ^4^	2.75	2.75	2.75	0.049	NS ^3^
Parity	2.60	2.20	3.40	0.436	NS ^3^
Milk (Kg/day)	29.20	29.80	29.50	2.157	NS ^3^

^1^ β-Hydroxybutyrate, ^2^ Days in Milk, ^3^ Not Significant, ^4^ Body Contition Score.

**Table 3:** Mean value (± SEM) plasma fatty acids analyzed in the four lipid classes (TLC-GC).

Nomenclature		Free Fatty Acids	Triglicerides	Cholesterol Esters	Phospholipids
**Short chain fatty acids (mg/dL)**					
C6	Caproic acid	/	0.073 ± 0.04	/	0.124 ± 0.33
C8	Caprylic acid	0.486 ± 0.58	13.242 ± 0.09	1.365 ± 0.54	0.041 ± 0.10
C10	Capric acid	0.178 ± 0.29	1.557 ± 0.17	0.764 ± 0.33	0.122 ± 0.10
C12	Lauric acid	0.818 ± 1.08	0.797 ± 0.61	2.618 ± 2.07	0.814 ± 1.28
**Long chain fatty acids (mg/dL)**					
C14	Myristic acid	0.309 ± 0.33	0.306 ± 0.32	1.141 ± 1.03	0.441 ± 0.59
C14:1ω5	Myristelaidic acid	0.066 ± 0.06	0.062 ± 0.05	0.678 ± 0.65	0.162 ± 0.28
C16	Palmitic acid	1.879 ± 1.70	1.573 ± 0.99	9.938 ± 5.42	14.133 ± 5.52
C16:1ω7	Palmitoleic acid	0.094 ± 0.16	0.035 ± 0.07	1.932 ± 1.31	0.369 ± 0.21
C17	Margaric acid	/	0.262 ± 0.23	0.174 ± 0.15	0.780 ± 0.39
C18	Stearic acid	1.556 ± 1.31	1.575 ± 0.98	1.542 ± 0.86	16.168 ± 6.10
C18:1ω9	Oleic acid	1.384 ± 2.98	0.315 ± 0.34	6.043 ± 4.13	10.015 ± 4.87
C18:1ω7	cis-Vaccenic acid	0.098 ± 0.17	0.047 ± 0.05	0.650 ± 0.36	1.282 ± 0.72
C18:2ω6	Linoleic acid	0.360 ± 0.30	0.326 ± 0.28	132.003 ± 92.83	20.719 ± 8.91
C18:3ω6	γ-Linolenic acid	0.015 ± 0.02	0.080 ± 0.12	2.417 ± 2.87	0.295 ± 0.22
C18:3ω3	α-Linolenic acid	0.018 ± 0.02	0.015 ± 0.02	9.826 ± 6.04	1.299 ± 0.74
C18:4ω3	Stearidonic acid	0.215 ± 0.18	0.261 ± 0.24	1.421 ± 1.55	0.343 ± 0.55
**Very long chain fatty acids (mg/dL)**					
C20	Arachidic acid	0.022 ± 0.04	0.050 ± 0.09	0.091 ± 0.09	0.101 ± 0.06
C20:1ω9	Gondoic acid	0.008 ± 0.02	0.005 ± 0.01	0.033 ± 0.08	0.084 ± 0.054
C20:1ω7	Paullinic acid	0.015 ± 0.02	0.018 ±0.02	0.078 ± 0.11	0.089 ± 0.062
C20:2ω6	Eicosadienoic acid	0.069 ± 0.15	0.153 ±0.26	1.368 ± 4.97	0.228 ± 0.20
)20:3ω9	Mead acid	0.024 ± 0.07	0.114 ±0.21	0.472 ± 1.97	0.062 ± 0.07
C20:3ω6	Dihomo-γ-linolenic acid	0.010 ± 0.01	0.016 ±0.02	0.261 ± 0.33	2.334 ± 1.42
C20:4ω6	Arachidonic acid	0.012 ± 0.03	0.008 ±0.01	1.373 ± 1.71	3.040 ± 1.45
C20:3ω3	Eicosatrienoic acid	0.009 ± 0.01	0.023 ±0.04	0.079 ± 0.19	0.031 ± 0.02
C20:4ω3	Eicosatetraenoic acid	0.157 ± 0.14	0.212 ±0.20	0.538 ± 0.58	0.380 ± 0.43
C20:5ω3	Eicosapentaenoic acid (EPA)	0.020 ± 0.04	0.007 ± 0.01	0.932 ± 0.75	0.575 ± 0.32
C22	Behenic acid	0.011 ± 0.02	0.020 ± 0.03	0.141 ± 0.19	0.712 ± 0.29
C22:1ω9	Erucic acid	0.006 ± 0.01	0.017 ± 0.02	0.042 ± 0.03	0.008 ± 0.01
C22:2ω6	Docosadienoic acid	0.009 ± 0.01	0.008 ± 0.03	0.056 ± 0.06	0.098 ± 0.19
C22:4ω6	Adrenic acid	0.050 ± 0.04	0.057 ± 0.06	0.212 ± 0.22	0.501 ± 0.34
C22:5ω3	Decosapentaenoic acid (DPA)	0.010 ± 0.02	0.007 ± 0.01	1.072 ± 7.20	1.678 ± 2.69
C22:6ω3	Docosahexaenoic acid (DHA)	0.020 ± 0.03	0.016 ± 0.05	0.082 ± 0.08	0.233 ± 0.30
C23	Tricosylic acid	0.007 ± 0.01	0.006 ± 0.01	0.017 ± 0.03	1.278 ± 0.65
C24	Lignoceric acid	0.016 ± 0.02	0.009 ± 0.02	0.151 ± 0.16	1.823 ± 6.24
C24:1ω9	Nervonic acid	0.034 ± 0.05	0.013 ± 0.03	0.090 ± 0.10	0.421 ± 0.35
C16 DMA	Dimethyl-acetal palmitic acid	0.365 ± 0.16	1.541 ± 4.74	5.649 ± 2.70	0.365 ± 0.28
Mg FA/dL	Mg of total FFA on dL	8.430 ± 7.33	22.845 ± 110.51	185.438 ± 99.82	81.166 ± 33.34
Mg/dL	Mg molecules on dL	8.430 ± 7.33	24.431 ± 119.15	434.604 ± 232.22	111.962 ± 45.90

**Table 4:** Plasma fatty acid mean (± SEM) related to the lipid class of Free Fatty Acids (TLC-GC).

Fatty Acids	BHB0 (mg/dL)	BHB1 (mg/dL)	BHB2 (mg/dL)	SEM	Correlation (*p*-Value)
C8	0.376	0.436	0.644	0.136	NS ^1^
C10	0.183	0.267	0.079	0.068	NS ^1^
C12	0.828	1.104	0.506	0.253	NS ^1^
C14	0.270	0.300	0.354	0.079	NS ^1^
C14:1 ω 5	0.061 ^AB^	0.038 ^A^	0.099 ^B^	0.012	0.002
C15	0.074	0.067	0.091	0.015	NS ^1^
C16	1.794	1.351	2.518	0.390	NS ^1^
C16:1 ω 7	0.109 ^B^	0.022 ^A^	0.156 ^B^	0.037	0.038
C18	1.482 ^AB^	0.981 ^A^	2.233 ^B^	0.289	0.012
C18:1 ω 9	0.863 ^A^	0.415 ^A^	2.900 ^B^	0.665	0.024
C18:1 ω 7	0.081 ^A^	0.033 ^B^	0.183^C^	0.038	0.020
C18:2 ω 6	0.361 ^B^	0.184 ^A^	0.544 ^B^	0.063	0.001
C18:3 ω 6	0.009 ^A^	0.012 ^AB^	0.022 ^B^	0.004	0.050
C18:3 ω 3	0.013 ^AB^	0.007 ^A^	0.033 ^B^	0.005	0.002
C18:4 ω 3	0.257 ^B^	0.113 ^A^	0.284 ^B^	0.040	0.007
C20	0.016	0.015	0.035	0.009	NS^1^
C20:1 ω 9	0.006 ^AB^	0.001 ^A^	0.018 ^B^	0.004	0.020
C20:1 ω 7	0.007 ^A^	0.010 ^A^	0.029 ^B^	0.005	0.003
C20:2 ω 6	0.078 ^AB^	0.002 ^A^	0.132 ^B^	0.033	0.024
C20:3 ω 9	0.044	0.022	0.008	0.016	NS ^1^
C20:3 ω 6	0.008 ^A^	0.008 ^A^	0.016 ^B^	0.002	0.028
C20:4 ω 6	0.019 ^A^	0.003 ^B^	0.014 ^B^	0.008	0.001
C20:3 ω 3	0.007	0.011	0.007	0.003	NS ^1^
C20:4 ω 3	0.182 ^B^	0.084 ^A^	0.209 ^B^	0.032	0.018
C20:5 ω 3	0.015	0.021	0.023	0.009	NS ^1^
C22	0.007 ^A^	0.007 ^A^	0.020 ^B^	0.004	0.019
C22:1 ω 9	0.006	0.006	0.005	0.002	NS ^1^
C22:2 ω 6	0.009	0.010	0.009	0.003	NS ^1^
C22:4 ω 6	0.050	0.060	0.039	0.010	NS ^1^
C22:5 ω 3	0.016	0.009	0.007	0.005	NS ^1^
C22:6 ω 3	0.034 ^A^	0.008 ^B^	0.018 ^AB^	0.002	0.013
C23	0.010	0.004	0.006	0.002	NS ^1^
C24	0.012	0.015	0.020	0.005	NS ^1^
C24:1 ω 9	0.026	0.041	0.035	0.012	NS ^1^
C16 DMA	0.382	0.338	0.378	0.037	NS ^1^
Mg FA/dL	7.694 ^AB^	6.008 ^A^	11.680 ^B^	1.665	0.050
Mg FFA/dL	7.694 ^AB^	6.008 ^A^	11.680 ^B^	1.665	0.050

^1^ not significant; ^A,B^ mean values with interparametric difference (*p* < 0.05).

**Table 5:** Plasma fatty acid mean (± SEM) related to the lipid class of Triglycerides (TLC-GC).

Fatty Acids	BHB 0 (mg/dL)	BHB 1 (mg/dL)	BHB 2 (mg/dL)	SEM	Correlation (*p*-Value)
C6	0.031	0.007	0.004	0.010	NS ^1^
C8	0.047	0.086	0.032	0.022	NS ^1^
C10	0.092	0.139	0.066	0.039	NS ^1^
C12	1.015	0.791	0.568	0.138	NS ^1^
C14	0.409 ^B^	0.342 ^AB^	0.167 ^A^	0.073	0.050
C14:1 ω 5	0.054 ^A^	0.052 ^A^	0.082 ^B^	0.009	0.040
C16	1.649	1.680	1.376	0.232	NS ^1^
C16:1 ω 7	0.032	0.017	0.060	0.015	NS ^1^
C17	0.393 ^B^	0.130 ^A^	0.291 ^AB^	0.047	0.001
C18	1.615 ^A^	1.708 ^A^	1.382 ^B^	0.130	0.042
C18:1 ω 9	0.347	0.244	0.366	0.080	NS ^1^
C18:1 ω 7	0.053 ^A^	0.041 ^B^	0.046 ^AB^	0.011	0.050
C18:2 ω 6	0.337	0.260	0.393	0.064	NS ^1^
C18:3 ω 6	0.040	0.087	0.109	0.027	NS ^1^
C18:3 ω 3	0.023	0.012	0.011	0.006	NS ^1^
C18:4 ω 3	0.342	0.177	0.282	0.055	NS ^1^
C20	0.084	0.034	0.035	0.021	NS ^1^
C20:1 ω 9	0.008	0.003	0.005	0.003	NS ^1^
C20:1 ω 7	0.020	0.015	0.019	0.005	NS ^1^
C20:2 ω 6	0.172	0.063	0.240	0.059	NS ^1^
C20:3 ω 9	0.139	0.075	0.137	0.049	NS ^1^
C20:3 ω 6	0.018	0.017	0.014	0.004	NS ^1^
C20:4 ω 6	0.004 ^A^	0.009 ^B^	0.012 ^B^	0.003	0.013
C20:3 ω 3	0.040 ^A^	0.016 ^B^	0.015 ^B^	0.009	0.038
C20:4 ω 3	0.229 ^AB^	0.129 ^A^	0.293 ^B^	0.045	0.035
C20:5 ω 3	0.005	0.005	0.012	0.003	NS ^1^
C22	0.019	0.014	0.028	0.008	NS ^1^
C22:1 ω 9	0.017 ^AB^	0.012 ^A^	0.023 ^B^	0.002	0.018
C22:2 ω 6	0.007	0.012	0.004	0.006	NS ^1^
C22:4 ω 6	0.042	0.049	0.082	0.013	NS ^1^
C22:5 ω 3	0.011	0.006	0.004	0.003	NS ^1^
C22:6 ω 3	0.010	0.029	0.007	0.012	NS ^1^
C23	0.006	0.005	0.007	0.002	NS ^1^
C24	0.006	0.010	0.010	0.004	NS ^1^
C24:1 ω 9	0.011	0.016	0.010	0.008	NS ^1^
C16 DMA	3.047	0.809	0.972	1.095	NS ^1^
Mg FA/dL	10.439	46.331	7.162	25.768	NS ^1^
Mg/dL	10.999	49.787	7.536	27.783	NS ^1^

^1^ not significant. ^A,B^ mean values with interparametric difference (*p* < 0.05).

**Table 6:** Plasma fatty acid mean (± SEM) related to the lipid class of Cholesterol Esters (TLC-GC).

Fatty Acids	BHB 0 (mg/dL)	BHB 1 (mg/dL)	BHB 2 (mg/dL)	SEM	Correlation (*p*-Value)
C8	1.521	1.301	1.273	0.134	NS ^1^
C10	0.760	0.780	0.747	0.085	NS ^1^
C12	3.313 ^A^	2.640 ^AB^	1.796 ^B^	0.504	0.050
C14	1.581	1.073	0.728	0.248	NS ^1^
C14:1 ω 5	0.489	0.822	0.697	0.162	NS ^1^
C16	8.969	10.334	10.506	1.367	NS ^1^
C16:1 ω 7	1.747	2.163	1.830	0.331	NS ^1^
C17	0.189	0.192	0.134	0.039	NS ^1^
C18	1.606	1.531	1.484	0.217	NS ^1^
C18:1 ω 9	5.884	6.325	5.839	1.047	NS ^1^
C18:1 ω 7	0.616	0.714	0.601	0.089	NS ^1^
C18:2 ω 6	115.731	159.321	113.527	22.931	NS ^1^
C18:3 ω 6	1.605	2.932	2.645	0.713	NS ^1^
C18:3 ω 3	11.922 ^B^	10.529 ^AB^	6.478 ^A^	1.428	0.036
C18:4 ω 3	1.675	1.347	1.230	0.392	NS ^1^
C20	0.098	0.082	0.095	0.023	NS ^1^
C20:1 ω 9	0.037	0.019	0.047	0.021	NS ^1^
C20:1 ω 7	0.125	0.050	0.062	0.027	NS ^1^
C20:2 ω 6	0.215	0.837	3.407	1.218	NS ^1^
C20:3 ω 9	0.045	0.157	1.386	0.480	NS ^1^
C20:3 ω 6	0.199	0.214	0.396	0.080	NS ^1^
C20:4 ω 6	1.488 ^AB^	0.735 ^A^	2.108 ^B^	0.409	0.047
C20:3 ω 3	0.066 ^AB^	0.130 ^A^	0.026 ^B^	0.046	0.050
C20:4 ω 3	0.649	0.346	0.672	0.141	NS ^1^
C20:5 ω 3	0.864 ^AB^	1.204 ^A^	0.641 ^B^	0.060	0.040
C22	0.133	0.183	0.092	0.048	NS ^1^
C22:1 ω 9	0.051	0.043	0.031	0.009	NS ^1^
C22:2 ω 6	0.048 ^AB^	0.080 ^A^	0.031 ^B^	0.013	0.032
C22:4 ω 6	0.229	0.209	0.195	0.055	NS ^1^
C22:5 ω 3	3.187	0.010	0.095	1.791	NS ^1^
C22:6 ω 3	0.071 ^AB^	0.109 ^A^	0.058 ^B^	0.020	0.039
C23	0.034 ^B^	0.011 ^AB^	0.005 ^A^	0.007	0.010
C24	0.148	0.120	0.089	0.039	NS ^1^
C24:1 ω 9	0.095	0.115	0.050	0.024	NS ^1^
C16 DMA	5.321	6.253	5.204	0.675	NS ^1^
Mg FA/dL	170.815	213.387	164.219	24.718	NS ^1^
Mg/dL	400.771	500.152	384.312	57.476	NS ^1^

^1^ not significant; ^A,B^ mean values with interparametric difference (*p* < 0.05).

**Table 7:** Plasma fatty acid mean (± SEM) related to the lipid class of Phospholipids (TLC-GC).

Fatty Acids	BHB 0 (mg/dL)	BHB 1 (mg/dL)	BHB 2 (mg/dL)	SEM	Correlation (*p*-Value)
C6	0.079	0.247	0.007	0.088	NS ^1^
C8	0.042	0.051	0.025	0.027	NS ^1^
C10	0.134	0.126	0.105	0.029	NS ^1^
C12	0.837	0.962	0.598	0.351	NS ^1^
C14	0.470	0.481	0.361	0.164	NS ^1^
C14:1 ω 5	0.226	0.104	0.180	0.076	NS ^1^
C16	14.999	13.168	14.593	1.510	NS ^1^
C16:1 ω 7	0.374	0.315	0.437	0.056	NS ^1^
C17	0.771	0.788	0.779	0.108	NS ^1^
C18	16.852	15.624	16.246	1.928	NS ^1^
C18:1 ω 9	10.508	8.953	10.948	1.322	NS ^1^
C18:1 ω 7	1.312	1.294	1.239	0.200	NS ^1^
C18:2 ω 6	22.604	19.674	20.344	2.438	NS ^1^
C18:3 ω 6	0.235	0.329	0.306	0.060	NS ^1^
C18:3 ω 3	1.435	1.283	1.193	0.203	NS ^1^
C18:4 ω 3	0.587	0.177	0.336	0.144	NS ^1^
C20	0.114	0.095	0.098	0.015	NS ^1^
C20:1 ω 9	0.070	0.090	0.088	0.015	NS ^1^
C20:1 ω 7	0.115	0.079	0.078	0.016	NS ^1^
C20:2 ω 6	0.034	0.067	0.196	0.048	NS ^1^
C20:3 ω 9	0.073	0.038	0.084	0.018	NS ^1^
C20:3 ω 6	1.988	2.562	2.355	0.386	NS ^1^
C20:4 ω 6	2.594	2.928	3.579	0.385	NS ^1^
C20:3 ω 3	0.035	0.028	0.032	0.006	NS ^1^
C20:4 ω 3	0.440	0.284	0.451	0.117	NS ^1^
C20:5 ω 3	0.547	0.578	0.598	0.087	NS ^1^
C22	0.766	0.628	0.772	0.078	NS ^1^
C22:1 ω 9	0.017 ^A^	0.004 ^B^	0.004 ^B^	0.003	0.018
C22:2 ω 6	0.034 ^A^	0.067 ^AB^	0.196 ^B^	0.048	0.048
C22:4 ω 6	0.587	0.396	0.559	0.090	NS ^1^
C22:5 ω 3	1.519	2.096	1.278	0.736	NS ^1^
C22:6 ω 3	0.280	0.280	0.127	0.081	NS ^1^
C23	1.426	1.055	1.431	0.172	NS ^1^
C24	1.002	3.069	0.951	1.702	NS ^1^
C24:1 ω 9	0.562	0.347	0.387	0.094	NS ^1^
C16 DMA	0.461 ^A^	0.295 ^B^	0.368 ^B^	0.025	0.049
Mg FA/dL	84.307	78.702	81.490	9.191	NS ^1^
Mg/dL	116.395	108.488	112.411	12.651	NS ^1^

^1^ not significant; ^A,B^ mean values with interparametric difference (*p* < 0.05).

**Table 8:** Predictive fatty acids with relative mean, median, minimum value, maximum value, and normal value of importance.

Fatty Acids	MeanImp ^1^	MedianImp ^2^	MinImp ^3^	MaxImp ^4^	NormHits ^5^	Decision
C20:1 ω 7 FFA	6.138	6.532	2.221	8.227	0.929	Confirmed
C20:2 ω 6 FFA	4.946	5.128	2.167	7.011	0.879	Confirmed
C18:2 ω 6 FFA	4.663	4.694	1.576	6.791	0.879	Confirmed
C18:1 ω 7 TG	5.273	5.564	1.382	7.377	0.859	Confirmed
C18:3 ω 6 FFA	4.829	4.981	1.224	6.763	0.848	Confirmed
C14:1 ω 5 TG	4.705	4.907	1.011	6.804	0.818	Confirmed
C14:1 ω 5 FFA	3.904	3.992	0.249	6.278	0.818	Confirmed
C18:3 ω 3 FFA	3.823	3.972	1.015	5.808	0.808	Confirmed
C20:3 ω 6 FFA	3.824	3.807	0.699	5.869	0.788	Confirmed
C22:0 FFA	3.602	3.743	1.062	5.628	0.727	Confirmed
C22:2 ω 6 CE	3.839	3.812	0.792	6.678	0.727	Confirmed
C20:3 ω 3 CE	3.734	3.898	0.740	6.012	0.697	Confirmed
C20:4 ω 3 TG	3.241	3.454	−0.154	5.703	0.687	Confirmed
C14:0 TG	3.611	3.716	0.146	5.896	0.687	Confirmed
C12:0 CE	3.381	3.436	0.386	6.048	0.667	Confirmed

^1^ average of the importance value; ^2^ median of the importance value; ^3^ minimum of the importance value; ^4^ maximum of the importance value; ^5^ value of normalized importance.

**Table 9:** Results related to the ROC of the predictive fatty acids for the development of hyperketonemia (Class BHB 2).

Fatty Acids	Cut Off (mg/dL)	AUC ^1^	Se ^2^	95% CI for Se	Sp ^3^	95% CI for Sp	*p*-Values
C20:1 ω 7 FFA	>0.0058	0.746	68.42	43.4–87.4	78.95	62.7–90.4	<0.0001
C20:2 ω 6 FFA	>0.0068	0.729	63.16	38.4–83.7	86.84	71.9–95.6	<0.0001
C18:2 ω 6 FFA	>0.437	0.726	63.16	38.4–83.7	86.84	71.9–95.6	<0.0001
C18:1 ω 7 TG	≤0.040	0.618	42.11	20.3–66.5	94.74	82.3–99.4	<0.0001
C18:3 ω 6 FFA	>0.026	0.724	57.89	33.5–79.7	86.84	71.9–95.6	<0.0001
C14:1 ω 5 TG	>0.097	0.514	47.37	24.4–71.1	84.21	68.7–94.0	<0.0001
C14:1 ω 5 FFA	>0.068	0.724	73.68	48.8–90.9	73.68	56.9–86.6	<0.0001
C18:3 ω 3 FFA	>0.032	0.706	42.11	20.3–66.5	94.74	82.3–99.4	<0.0001
C20:3 ω 6 FFA	>0.011	0.715	68.42	43.4–87.4	73.68	56.9–86.6	<0.0001
C22:0 FFA	>0.019	0.634	42.11	20.3–66.5	89.47	75.2–97.1	<0.0001
C22:2 ω 6 CE	≤0.046	0.733	78.95	54.4–93.9	73.68	56.9–86.6	<0.0001
C20:3 ω 3 CE	≤0.040	0.731	84.21	60.4–96.6	57.89	40.8–73.7	<0.0001
C20:4 ω 3 TG	>0.311	0.637	57.89	33.5–79.7	78.95	62.7–90.4	<0.0001
C14:0 TG	≤0.226	0.701	89.47	66.9–98.7	55.26	38.3–71.4	<0.0001
C12:0 CE	≤1.093	0.738	57.89	33.5–79.7	86.84	71.9–95.6	<0.0001

^1^ Area Under the Curve; ^2^ Epidemiologic Sensitivity; ^3^ Epidemiologic Specificity.
